# Long-Term Outcomes of Maxillary Alveolar Process Trauma and Primary Incisor Injury in Early Childhood: A Case Report

**DOI:** 10.3390/jcm14103275

**Published:** 2025-05-08

**Authors:** Sanja Vujkov, Stojan Ivic, Bojan Petrovic, Duska Blagojevic, Isidora Neskovic, Ana Tadic, Jelena Komsic

**Affiliations:** Department of Dental Medicine, Faculty of Medicine, University of Novi Sad, 21000 Novi Sad, Serbia; sanja.vujkov@mf.uns.ac.rs (S.V.); stojan.ivic@mf.uns.ac.rs (S.I.); bojan.petrovic@mf.uns.ac.rs (B.P.); duska.blagojevic@mf.uns.ac.rs (D.B.); ana.tadic@mf.uns.ac.rs (A.T.); jelena.komsic@mf.uns.ac.rs (J.K.)

**Keywords:** tooth injuries, alveolar process, tooth eruption, deciduous tooth, permanent tooth, developmental defects of enamel

## Abstract

**Background**: Traumatic injuries to the alveolar process and primary teeth in early childhood can have long-term consequences on the development of permanent dentition and eruption pathways. **Objective**: This case report aims to illustrate the impact of early orofacial trauma on the eruption and development of permanent maxillary incisors and to emphasize the importance of timely interdisciplinary management. **Case Presentation**: An 8-year-old female patient presented to a pediatric dentistry clinic with delayed eruption of the maxillary anterior permanent teeth. In contrast, her monozygotic twin sister exhibited complete eruption of all permanent anterior teeth, raising parental concern regarding a possible pathological delay. Her medical history revealed orofacial trauma at the age of two, resulting in an alveolar process fracture, avulsion of the primary maxillary left central incisor (tooth 61), and luxation of the primary maxillary right central incisor (tooth 51). A clinical examination demonstrated sufficient arch space without signs of eruption and enamel defects on tooth 52. Radiographic evaluations, including panoramic imaging and cone beam computed tomography (CBCT), confirmed the presence of impacted permanent teeth with structural anomalies suggestive of trauma-related developmental disturbances. Results: The patient underwent a multidisciplinary treatment over a three-year period involving pediatric dentistry, oral surgery, and orthodontics. Management included surgical exposure of the impacted teeth followed by orthodontic traction to guide the eruption and treatment of enamel hypoplasia. **Conclusions**: This case highlights the long-term consequences of early traumatic dental injuries on permanent dentition development. It underscores the necessity of early diagnosis and a coordinated interdisciplinary approach to optimize outcomes and enhance the long-term oral health and quality of life of affected individuals.

## 1. Introduction

The alveolar process of the maxilla plays a crucial role in both dental and facial development, providing essential support for primary and permanent teeth. Although relatively rare, fractures of this region in early childhood can lead to significant short- and long-term consequences, impacting both dentition and maxillofacial growth [1]. Due to the plasticity of developing bones and the close anatomical relationship between primary teeth and developing permanent tooth buds, trauma to the maxillary alveolar process can result in a wide range of complications [2]. These include delayed eruption, impaction, structural anomalies, and disturbances in occlusion and maxillary growth [3].

Facial trauma in early childhood most commonly results from falls, play-related accidents, and less frequently, non-accidental injuries. While the maxilla demonstrates some resilience due to its immature structure, direct impacts can still cause alveolar fractures, often accompanied by dental injuries such as avulsion, luxation, or the intrusion of primary teeth [4]. Research shows that trauma to the primary dentition can significantly affect the development of permanent teeth, as tooth germs are situated in close proximity to the roots of the primary teeth [5]. The incidence of alveolar fractures in pediatric patients is variable, with estimates suggesting they represent 14.1% of all facial fractures in children [6]. The pathophysiological response to alveolar fractures in young children is influenced by the high vascularity and regenerative capacity of the maxilla. However, despite the potential for spontaneous healing, complications may occur due to disrupted eruption pathways of permanent teeth, damage to enamel and dentin formation, or the malalignment of fractured bone segments [7]. The prognosis is determined by factors such as the severity of trauma, the degree of displacement, and the involvement of primary teeth. While minor fractures may resolve without intervention, more severe cases often require a multidisciplinary approach to prevent long-term functional and esthetic issues [8].

One of the most significant complications of alveolar fractures in early childhood is the delayed eruption of permanent teeth. This may result from physical obstructions such as bone fragments or fibrotic tissue, ankylosis, or the disruption of the eruption mechanism. Additionally, impacted teeth may develop structural anomalies such as enamel hypoplasia, dilaceration, or altered root morphology [9]. In more severe cases, trauma can lead to arrested root development or the premature closure of the apical foramen, potentially compromising the long-term prognosis of the affected teeth [10].

Malocclusion is another important concern following alveolar trauma. If fractured segments are not properly realigned, displacement can result in crossbite, open bite, or dental arch asymmetry, often requiring early orthodontic interventions to re-establish proper occlusion and function [11]. Furthermore, alveolar fractures may influence maxillary growth, especially if the trauma involves sutural areas. Consequently, long-term follow-up is necessary to monitor for growth disturbances that could require orthopedic or surgical correction [12].

Early and accurate diagnosis is essential for the effective management of alveolar fractures and the prevention of complications. Clinical examinations should assess tooth mobility, displacement, and pulp vitality. Radiographic imaging, including panoramic radiographs, periapical X-rays, and cone beam computed tomography (CBCT), provides critical information about the extent of the fracture, the condition of developing permanent teeth, and potential obstructions to eruption. In complex cases, three-dimensional imaging aids in treatment planning and determining the need for surgical intervention [13].

Given the complexity of alveolar fractures in young children, a multidisciplinary treatment strategy involving pediatric dentists, oral and maxillofacial surgeons, and orthodontists is often required. Initial management focuses on stabilizing the fractured segments, repositioning displaced teeth, and preventing infection [12]. When delayed eruption or tooth impaction occurs, surgical exposure followed by orthodontic traction may be necessary to guide the teeth into their correct position. Close and continuous follow-up is crucial to monitor healing and to promptly address any emerging complications [14].

While alveolar fractures comprise approximately 14.1% of facial fractures in pediatric patients [6,7], the prevalence of subsequent developmental disturbances in permanent successors ranges widely. Studies have reported such complications—including enamel hypoplasia, crown/root malformations, or delayed eruption—in 12–69% of cases involving trauma to the primary dentition [3,4]. However, isolated cases of delayed eruption, particularly without ankylosis or pathological obstructions, remain rare in the literature. The present case is further distinguished by the presence of a monozygotic twin with normal eruption, providing a unique internal control that highlights the likely causal role of early trauma. The aim of this case report is to illustrate the long-term impact of early orofacial trauma on the eruption and development of permanent maxillary incisors, specifically highlighting the delayed eruption and structural anomalies that can result from trauma to primary teeth. Through a comparison with a monozygotic twin, this report underscores the importance of early diagnosis, careful monitoring, and an interdisciplinary approach to managing such cases to optimize outcomes and prevent long-term complications in affected individuals.

## 2. Case Presentation

**Figure 1:**
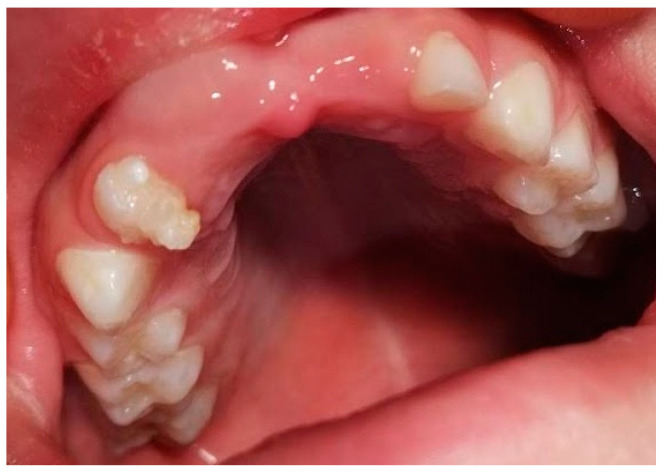
Intraoral view of dental arch and structurally changed tooth 52 at February 2022.

An 8-year-old girl, accompanied by her parents, was referred to the Department of Pediatric Dentistry, Clinic of Dentistry of Vojvodina, Novi Sad, Serbia, due to delayed eruption of the maxillary permanent anterior teeth (Figure 1).

**Figure 2:**
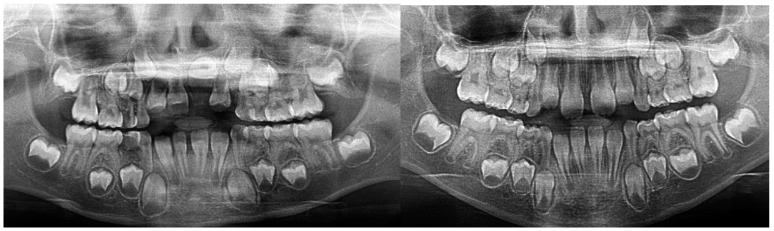
Panoramic radiography images taken during the first visit of the patient and her twin sister in February 2022.

**Figure 3:**
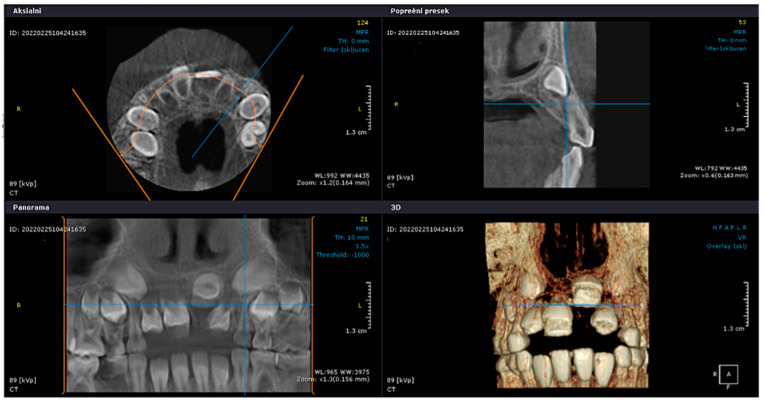
CBCT February 2022.

The patient presented alongside her monozygotic twin sister, whose permanent maxillary anterior teeth had fully erupted. The clinical examination revealed adequate arch space in the anterior maxilla, but no clinical signs of eruption. In addition, tooth 52 exhibited structural enamel abnormalities. Radiographic assessments, including panoramic imaging and cone beam computed tomography (CBCT), confirmed the presence of impacted permanent maxillary incisors, with structural changes indicative of trauma-induced developmental disturbances. Orthopantomographic (OPG) imaging was performed for both sisters (Figure 2), and the extraction of primary tooth 52 was carried out. The initial CBCT scan revealed the abnormal position of the tooth germ of 21, the undeveloped root formation of tooth 21, and enamel structural irregularities affecting teeth 12, 11, 21, and 22 (Figure 3).

**Figure 4:**
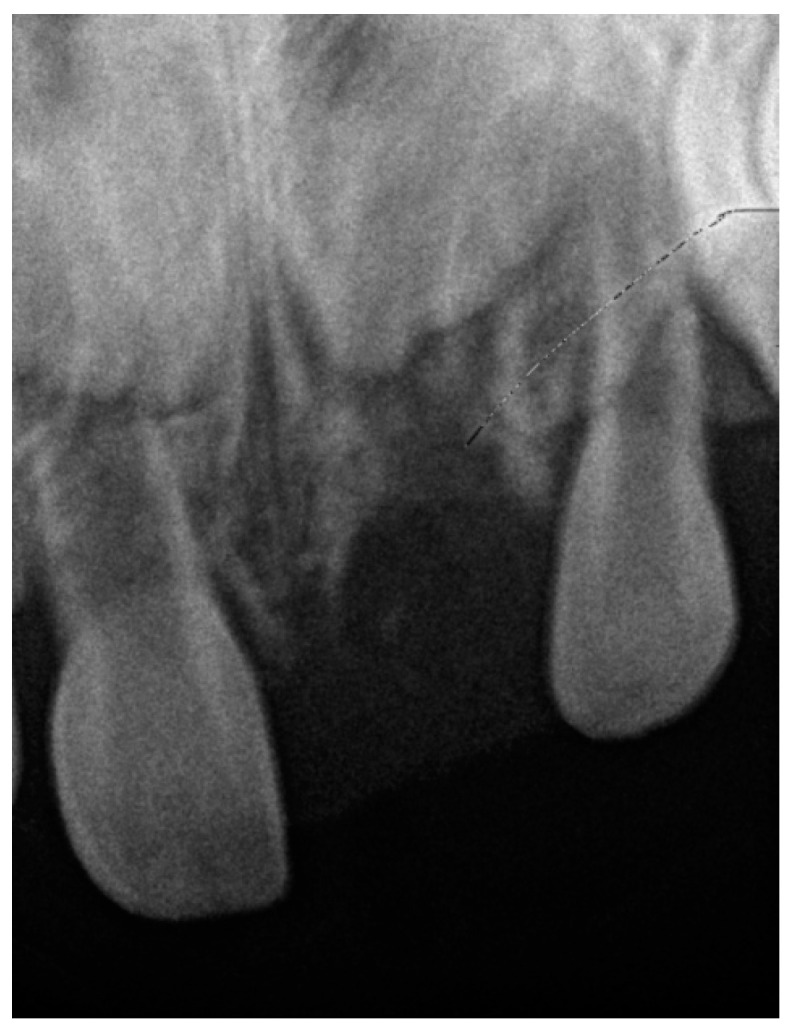
Radiography of traumatized primary teeth in 2016.

A review of the patient’s medical records from 2016 revealed a retroalveolar radiograph taken at the age of 11 months, following a dental trauma. The radiograph (Figure 4) showed an empty alveolus corresponding to the avulsed primary tooth 61, an enlarged periodontal ligament space around tooth 51 (indicating luxation), and the permanent tooth germs 11 and 21 positioned at the same level. The dental report from that time described a suspected fracture of the maxillary alveolar process.

A thorough review of the patient’s medical history revealed a significant traumatic event during early childhood. At the age of 11 months, the patient fell from a baby walker, striking her mouth against a hard surface. Immediately following the fall, bleeding from the oral cavity was observed, prompting the parents to seek emergency care. She was referred to the pediatric dentist at the same clinic where she is currently being treated. A comprehensive dental examination and radiographic evaluation were performed. The radiographs revealed the avulsion of the primary maxillary left central incisor (tooth 61), luxation of the primary maxillary right central incisor (tooth 51), and a suspected fracture of the maxillary alveolar process (Figure 4). The radiograph showed an empty alveolus corresponding to the avulsed primary tooth 61, an enlarged periodontal ligament space around tooth 51 (indicating luxation), and the permanent tooth germs 11 and 21 positioned at the same level. An oral surgeon was consulted, but due to the patient’s young age and limited cooperation, no immediate therapeutic procedures could be undertaken. The parents were advised on oral hygiene and dietary measures, and the patient was asked to attend regular follow-ups for the next sixth months.

**Figure 5:**
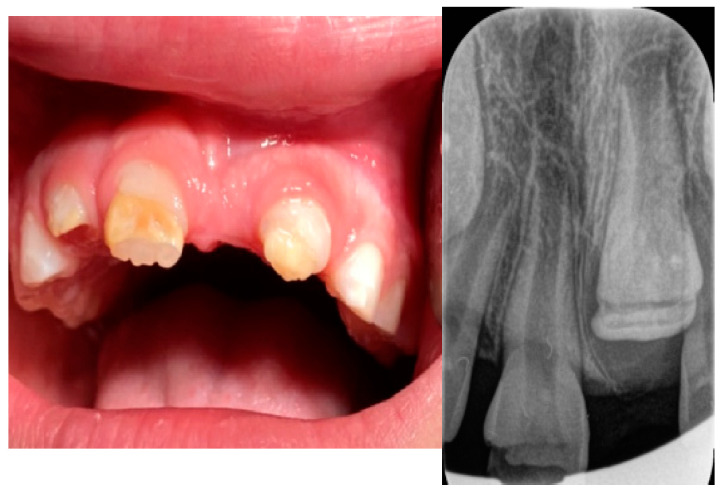
Intraoral view and radiography in March 2023.

The patient was managed using a multidisciplinary approach, involving a pediatric dentist, oral surgeon, and orthodontic specialist. We decided to postpone the surgical-orthodontic intervention until there was further root development in tooth 21. At a one-year follow-up, the eruption of teeth 12, 11, and 22 was noted, although they exhibited enamel defects in the form of a horizontal hypoplastic line on their vestibular surfaces. CBCT and radiographic imaging showed the initial stages of root formation of tooth 21 (Figure 5).

**Figure 6:**
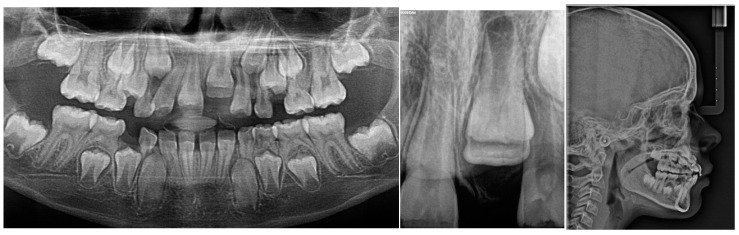
Radiography in April 2024.

**Figure 7:**
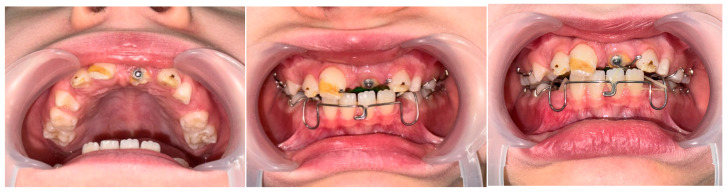
Orthodontic treatment of erupted tooth 21 in January 2025.

**Figure 8:**
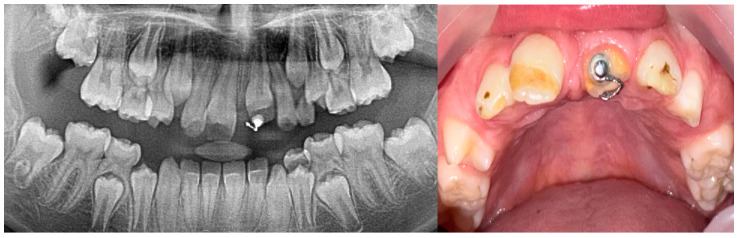
Radiographic findings and appearance of the erupted tooth in April 2025.

After two years, a clinical evaluation confirmed that tooth 21 had still not erupted, despite advanced root development visible via imaging. The tooth was now positioned more occlusally compared to its previous location (Figure 6). Consequently, surgical-orthodontic therapy was initiated. Due to the patient’s age and mixed dentition, removable appliances were used to apply traction to the upper left central incisor and maintain the space for its eruption. As for the traction, the lower plate was used as an anchor in the antagonistic jaw, to ensure a longer elastic effect of the light elastics given to the patient. The patient’s parents were instructed to change the elastics after a 48 h period. The upper plate was used to open and maintain space for the tooth. After the completion of permanent dentition, as well as the preparation of the hypoplastic surfaces of the enamel, orthodontic therapy with fixed appliances is planned to optimize the occlusion, as well as the patient’s aesthetic appearance. At the subsequent follow-up, the eruption of tooth 21 was observed (Figure 7), and orthodontic treatment was continued to align the dentition properly. At the follow-up examination in April 2025, the advanced root development of tooth 21 was observed, along with a more pronounced stage of tooth eruption (Figure 8).

The initial trauma to the alveolar process not only disrupted the eruption trajectory of the permanent successor teeth but also induced structural enamel defects, clinically evident as a horizontal hypoplastic band affecting all four maxillary incisors. Management to date has consisted of the application of topical remineralization agents, including fluoride and casein phosphopeptide-amorphous calcium phosphate (CPP-ACP) formulations. While esthetic rehabilitation with composite resin restorations is planned to address the hypomineralized enamel, the patient’s parents have, to date, declined authorization for restorative treatment. At the most recent follow-up, the hypoplastic enamel areas remained clinically stable, with no evidence of progressive enamel wear or loss. Occlusal function was preserved, and no signs of functional impairment were observed. Both the patient and parents reported satisfaction with her current esthetic appearance, despite the presence of enamel defects. Standardized clinical photographs documenting the present condition of the maxillary incisors have been included to illustrate these findings.

## 3. Discussion

In the present case, early childhood trauma to the primary maxillary incisors and alveolar process led to multiple complications, including delayed eruption, altered eruption pathways, and developmental anomalies of the permanent successor teeth. This clinical presentation is consistent with findings from previous studies, which have demonstrated that trauma to the primary dentition can significantly disrupt the development of underlying permanent teeth [15,16]. These disturbances may arise from direct damage to the permanent tooth germ, or as a result of secondary factors such as ankylosis, fibrotic scarring, or pathological changes in the eruption trajectory [17,18]. The initial trauma, which occurred at the age of 11 months, resulted in the avulsion of tooth 61, luxation of tooth 51, and a suspected alveolar fracture—all of which were confirmed through early radiographic imaging and clinical documentation. The close anatomical relationship between the roots of primary teeth and the developing germs of permanent successors is a well-established risk factor in such cases [19,20]. Particularly, injuries such as the intrusion and avulsion of primary incisors have been linked to enamel hypoplasia, root malformation, dilacerations, and the impaction of permanent incisors [21]. In our patient, the delayed eruption of tooth 21 persisted for over two years following the eruption of the adjacent incisors and was accompanied by hypoplastic enamel bands across all four maxillary permanent incisors. These structural anomalies are consistent with trauma-induced disturbances of the ameloblast layer during crown formation. The horizontal hypoplastic lines observed clinically correspond to the period of trauma and highlight the chronological nature of enamel disruption during odontogenesis [16].

Both biological and mechanical factors contributed to the delayed eruption observed. Trauma to Hertwig’s epithelial root sheath likely impaired root development of tooth 21, while mechanical obstructions, such as fibrotic tissue formation or alveolar bone displacement, may have altered the eruption pathway [20,22]. The abnormal position of the tooth germ on CBCT, delayed root maturation, and the lack of spontaneous eruption, despite adequate arch space, support the hypothesis of both biological and mechanical interference. Cone beam computed tomography (CBCT) proved essential in this case, allowing for a detailed assessment of root morphology, tooth positioning, and surrounding alveolar structures. The recent literature has increasingly highlighted CBCT as a superior diagnostic tool compared to conventional radiographs, particularly for detecting ankylosis, root dilaceration, and ectopic eruption pathways in complex trauma cases [13]. By providing cross-sectional imaging and three-dimensional reconstructions, CBCT offers a more accurate depiction of anatomical details and radiolucencies. It also has clear advantages over periapical radiography in diagnosing alveolar fractures and luxations; two-dimensional radiographs often fail to reveal the true extent of injury, especially since most displacements in alveolar process fractures occur in the sagittal plane [23,24].

Early detection of such complications remains challenging due to their delayed clinical manifestation, reinforcing the importance of long-term follow-up after early childhood dental trauma [19]. The management of this case required a coordinated multidisciplinary approach, involving pediatric dentistry, oral surgery, and orthodontics. The initial strategy involved monitoring root development, followed by surgical exposure and the orthodontic traction of the impacted tooth once an adequate root length was achieved. This approach is supported in the literature, which emphasizes that early surgical-orthodontic intervention improves the successful eruption and positioning of the tooth, while reducing the need for more extensive future interventions [25,26].

Despite the successful eruption and alignment of tooth 21, residual esthetic concerns remain due to enamel hypoplasia, which was managed non-invasively with topical remineralization therapy. The parents declined composite restorations, underscoring the importance of engaging families in shared decision making and providing comprehensive counseling on long-term esthetic outcomes.

The prognosis in such cases is largely dependent on the extent of the initial trauma and the timeliness of the intervention. As observed, the consequences of early dental trauma are not limited to eruption disturbances, but may also affect crown morphology, root development, occlusion, and even maxillofacial growth [14]. Studies have highlighted long-term risks such as malocclusion, tooth loss, and esthetic compromise, all of which may impact masticatory function, speech, and psychosocial well-being, particularly during the formative adolescent years [27,28].

The management of the delayed eruption of permanent teeth can involve non-surgical approaches, such as space maintenance and the use of eruption guidance appliances, particularly when there is a favorable likelihood of eruption [18]. However, when spontaneous eruption fails or the tooth is malpositioned, surgical exposure combined with orthodontic traction becomes necessary. The timing of surgical intervention is critical; early exposure can facilitate eruption and simplify orthodontic alignment, whereas delayed exposure may be advantageous for allowing further root development and better periodontal outcomes [27]. In the present case, due to the unfavorable position and lack of spontaneous eruption, surgical-orthodontic intervention was selected to optimize both functional and esthetic results.

In considering differential diagnoses, molar incisor hypomineralization (MIH) was evaluated but excluded, since the case did not meet the criteria for MIH as listed in the newest guidelines [29]. MIH typically affects the first permanent molars and incisors symmetrically and is associated with systemic factors during the first years of life. In this case, enamel defects were limited to the maxillary central and lateral incisors directly associated with the traumatic incident, with no involvement of molars or other teeth. Additionally, the monozygotic twin sister showed no enamel anomalies, further supporting a trauma-induced etiology rather than a systemic developmental disturbance.

Given these multifactorial implications, clinicians must maintain a high degree of suspicion when managing patients with a history of trauma to primary teeth. Thorough clinical and radiographic evaluations, early CBCT imaging in selected cases, and individualized treatment plans are key to mitigating complications [24,30]. Long-term follow-up, spanning the entire mixed dentition phase, is essential to ensure optimal outcomes [25].

This case report has several limitations. First, radiographic monitoring was constrained by the need to minimize radiation exposure in a pediatric patient, which limited the frequency of imaging. Second, the timing of interventions was influenced by clinical judgment regarding the patient’s root development and eruption potential, introducing variability that may not be generalizable. Third, as this case involved a unique traumatic event in early childhood, the findings may not be extrapolated to all patients with delayed eruption or enamel defects. Larger cohort studies are needed to validate the observations reported here.

Future research should aim to establish predictive models correlating trauma severity with eruption disturbances and to explore regenerative therapies for damaged tooth germs and alveolar structures [31]. Additionally, there is a growing need to investigate the psychological impact of trauma-induced dental anomalies on children and adolescents, with the goal of enhancing patient-centered care and improving their quality of life [32].

## 4. Conclusions

Fractures of the maxillary alveolar process in early childhood pose significant challenges due to their potential to disrupt normal dental development and maxillofacial growth. While the regenerative potential of young bone is high, complications such as delayed eruption, dental anomalies, and malocclusion require careful monitoring and intervention. Early diagnosis and a well-coordinated multidisciplinary approach are essential in minimizing long-term consequences and ensuring optimal functional and esthetic outcomes for affected patients. Future research should focus on developing improved diagnostic and treatment protocols to enhance the management of these injuries and prevent their associated complications.

## Data Availability

Data are contained within the article.
